# Is Additive Manufacturing an Environmentally and Economically Preferred Alternative for Mass Production?

**DOI:** 10.1021/acs.est.2c04927

**Published:** 2023-04-17

**Authors:** Sangjin Jung, Levent Burak Kara, Zhenguo Nie, Timothy W. Simpson, Kate S. Whitefoot

**Affiliations:** ○ Mechanical, Aerospace, and Materials Engineering, Southern Illinois University, Carbondale, Illinois 62901, United States; ‡ Mechanical Engineering, Carnegie Mellon University, Pittsburgh, Pennsylvania 15213, United States; § Mechanical Engineering, Tsinghua University, Beijing 100084, China; ¶ Mechanical Engineering, Pennsylvania State University, University Park, Pennsylvania 16802, United States; # Engineering and Public Policy, Carnegie Mellon University, Pittsburgh, Pennsylvania 15213, United States

**Keywords:** 3D printing, additive manufacturing, greenhouse gas emissions, industrial decarbonization, green production

## Abstract

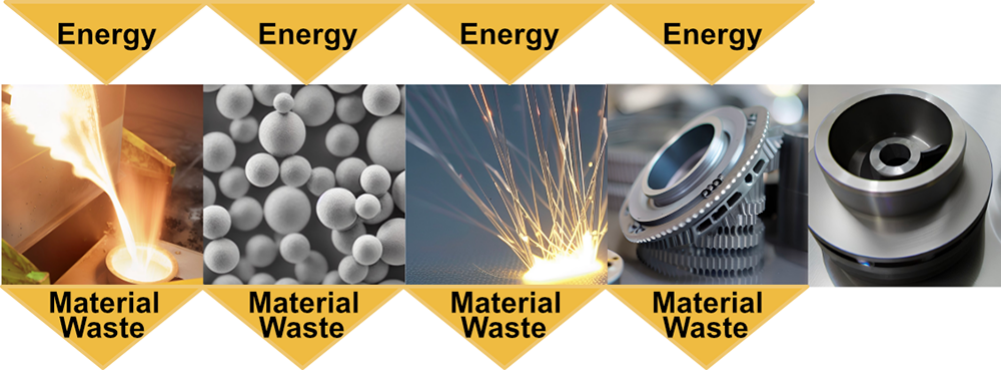

The manufacturing sector accounts for a large percentage of global energy use and greenhouse gas emissions, and there is growing interest in the potential of additive manufacturing (AM) to reduce the sector’s environmental impacts. Across multiple industries, AM has been used to reduce material use in final parts by 35–80%, and recent publications have predicted that AM will enable the fabrication of customized products locally and on-demand, reducing shipping and material waste. In many contexts, however, AM is not a viable alternative to traditional manufacturing methods due to its high production costs. And in high-volume mass production, AM can lead to increased energy use and material waste, worsening environmental impacts compared to traditional production methods. Whether AM is an environmentally and economically preferred alternative to traditional manufacturing depends on several hidden aspects of AM that are not readily apparent when comparing final products, including energy-intensive and expensive material feedstocks, excessive material waste during production, high machine costs, and slow rates of production. We systematically review comparative studies of the environmental impacts and costs of AM in contrast with traditional manufacturing methods and identify the conditions under which AM is the environmentally and economically preferred alternative. We find that AM has lower production costs and environmental impacts when production volumes are relatively low (below ∼1,000 per year for environmental impacts and below 42–87,000 per year for costs, depending on the AM process and part geometry) or the parts are small and would have high material waste if traditionally manufactured. In cases when the geometric freedom of AM enables performance improvements that reduce environmental impacts and costs during a product’s use phase, these can counteract the higher production impacts of AM, making it the preferred alternative at larger production volumes. AM’s ability to be environmentally and economically beneficial for mass manufacturing in a wider variety of contexts is dependent on reducing the cost and energy intensity of material feedstock production, eliminating the need for support structures, raising production speeds, and reducing per unit machine costs. These challenges are not primarily caused by economies of scale, and therefore, they are not likely to be addressed by the increasing expansion of the AM sector. Instead, they will require fundamental advances in material science, AM production technologies, and computer-aided design software.

## Introduction

Almost one-quarter of global greenhouse gas emissions and over one-third of global energy consumption is caused by industry, with the vast majority due directly or indirectly to the manufacturing sector.^1−3^ Additive manufacturing (AM) has been recognized as a potentially disruptive technology that could dramatically reduce the environmental impacts of manufacturing.^4−6^ Across multiple industries, AM has been used to reduce material use in final parts by 35–80%,^4,7^ and recent publications have predicted that AM will enable the fabrication of customized products locally and on-demand, reducing shipping and material waste.^5,6^

Is AM, however, a viable environmentally preferable alternative to traditional manufacturing methods? And will it become so in the future? The answers depend on several hidden aspects of AM that are not readily apparent when comparing the final form of objects produced with either AM or traditional manufacturing processes. These include the energy intensity of material inputs, material waste during production, and build rates. Additionally, AM faces challenges that may make it economically prohibitive for certain applications even as the technology increases in scale, and so, assessment of the sustainability of AM should consider both economic and environmental considerations.

This Critical Review assesses the economic and environmental impacts of AM in comparison with traditional manufacturing methods, such as machining, pressing, and injection molding, and identifies the conditions under which AM is preferred. We find that the prospect for AM to create distributed manufacturing that would supply locally sourced customized products is limited by the high costs of AM machines and slow production rates. Because the highest cost machine components are already produced at large scale to supply other industries, this barrier will not be reduced by increasing the scale of AM; rather, it will require technological changes in AM processes. Our review finds that, across a wide variety of materials and part geometries, AM has lower environmental impacts than traditional manufacturing processes when production volumes are very low (approximately 1,000 parts per year or less) and the part geometry has a solid-to-envelope ratio of less than 1:7.^7,8^ We find similar results for the production costs of AM relative to traditional manufacturing although the break-even production volume differs (ranging from 42 to 87,000 parts per year or less depending on the AM modality and part geometry^9−14^). In cases where AM can shorten supply chains or enable part geometries that provide sufficient performance improvements during the product’s use, such as when lighter weight parts reduce fuel consumption in automotive and aerospace applications, these can counteract the higher production costs and environmental impacts, making AM preferable at larger production volumes.

We review emerging advances in material science, AM production technologies, and design optimization that could change the equation for AM and make it the lower cost and lower environmental-impact alternative for mass manufacturing in a wider variety of areas. We find that environmental and economic sustainability are synergistic for AM: advances that improve the environmental impacts of AM also improve production costs. Developing AM technologies and optimization techniques that eliminate support structures that contribute to material waste, new approaches to reduce AM machine and postprocessing costs, and material production methods that are cheaper and less energy intensive could make AM the economically and environmentally preferred choice for many mass manufacturing applications in the future.

## Common AM Processes

When a part is produced using AM, material feedstock, often in the form of a powder, wire, or liquid, is deposited and solidified to create a solid part. There are many different AM processes, defined by the type of material feedstock and the form, energy, and sequence of deposition and solidification. As of 2020, over 2.1 million units of AM machines were globally shipped with material extrusion and vat photopolymerization constituting the largest shares, and powder bed fusion (PBF) and directed energy deposition (DED) are anticipated to grow in the future.^15,16^

Material extrusion feeds melted plastic such as ABS and PLA through one or more nozzles and uses a layer-by-layer deposition and cooling process that is relatively simple and less expensive than other AM processes.^17^ Vat photopolymerization solidifies liquid photopolymer using UV lasers, which can produce thin and fine shapes of parts.^18^ DED and PBF, on the other hand, deposit metallic feedstock (e.g., powder or wire) that is heated together to form an object. PBF lays down a volume of powder metal in an enclosed chamber and selectively heats the powder into a 3D part, most commonly with electron beam melting (EBM) or laser-based systems such as Direct Metal Laser Sintering (DMLS). DED directly deposits feed material (either powder or wire) and melts the material together using a laser, electron beam, or plasma arc. Wire-fed or powder-fed DED can produce parts with much larger size than other AM modalities, but PBF offers higher resolution when making metal parts compared to DED.^19^

## Economic and Environmental Advantages of AM

### Environmental Advantages of AM

An advantage of AM processes is that they do not require tooling to form parts into their desired shape, unlike many traditional manufacturing methods such as injection molding, die casting, forging, and stamping. The elimination of tooling reduces environmental impacts by eliminating environmental emissions and waste embodied in the tooling supply chain. For example, injection molding requires high energy consumption to manufacture molds. Using AM eliminates this energy consumption, significantly reducing the greenhouse-gas emissions for the lifecycle of products.^7,20^

A second advantage of AM is that its geometric freedom has the potential to reduce environmental impacts when supply chains can be shortened to produce customized products or when significantly less material is wasted compared to traditional manufacturing. For example, Vallourec has collaborated with RAMLAB in the port of Rotterdam to produce replacement parts of a waterbushing on site for maintenance rather than shipping in the products.^21^ The weight of the final part is half that of the traditionally manufactured part, and they have reduced emissions by 45% compared to conventional machining and forging processes.

### Production Cost Advantages of AM

The ability to eliminate tooling and have increased geometric freedom with AM is a benefit not only for environmental impacts but also for production costs. In traditional manufacturing processes, the tooling itself has to be machined, and it often comprises a large percentage of production costs. For example, for injection molding or die casting, tooling costs can be over 80% of total production costs.^10,11^ AM eliminates these costs.

The elimination of tooling and the geometric freedom of AM allows it to produce a variety of parts without substantially increasing unit costs. This contrasts strongly with molding, pressing, and stamping in which a fixed geometry must be produced at high production volume in order to overcome the tooling costs, and the geometry must be fixed early in the product design process to begin manufacturing the tooling.^11^

The geometric freedom of AM enables customization of product size and shape to suit heterogeneous customer preferences and allows manufacturers to much more rapidly pivot product designs to reflect changes in market demand.^22^ It also enables production of complex parts that would require high-cost finishing operations using traditional manufacturing methods. AM has economic advantages over machining processes when a part has high structural complexity.^23^ For example, geometries such as lattice structures, which create lightweight yet strong parts, are very difficult to produce with traditional manufacturing methods and generally require assembly of many separate components^24^ or require their shapes to be optimized for traditional manufacturing methods (such as CNC) that introduce additional manufacturability constraints, making the final parts bulkier compared to their AM counterparts.^25−27^ With AM, they can be produced relatively easily as one solid structure within the same process.

## Hidden Economic and Environmental Costs of AM

While AM has the potential to reduce environmental impacts and production costs, it can also increase them due to energy-intensive and costly material inputs, material waste during production, high machine costs, and slow rates of production. We discuss the factors that can cause AM materials, machines, and processes to increase production costs and environmental impacts of AM relative to traditional manufacturing methods.

### Environmental Disadvantages of AM

AM material feedstock such as metal and polymer powders is more energy intensive than those in traditional manufacturing processes because the material production processes require additional process steps to fabricate the material, and, particularly for metal powder production, the processes can consume large amounts of energy.^7,13^ For example, AM metal powder is produced by atomization processes where metal ingot, billet, or wire is fed into a furnace or chamber and broken down into small drops of liquid metal using water, gas, or plasma, which cool into powder.^28^ Material production for powder-fed AM has the highest energy consumption and environmental impacts compared to other product lifecycle stages from cradle to gate.^29−34^ The higher energy consumption increases damages to human health, ecosystems, and resource availability.^35^ Relatively high reject rates in AM production compared to traditional manufacturing^4^ further increase the overall environmental impacts associated with material production because of the lost material.

The slow production rate of AM is another disadvantage that increases environmental impacts because of the energy consumption necessary to run the AM machines for this amount of time. Producing an aeronautical turbine using EBM-PBF requires 15.6 h per part as opposed to only 5.9 h using milling. As a result, EBM-PBF consumes 25% more energy than milling (from 27.5 to 34.4 kWh).^36^ Many AM processes also require relatively longer postprocessing operations to remove support structures and smooth the surface finish and perform additional postprocessing steps such as heat treatment, wire electrical discharge machining (EDM), hot isostatic pressing (HIP), and shot peening.^4^ An increase in postprocessing leads to additional environmental impacts. Faludi et al. showed that adding a wire EDM process for postprocessing increases energy consumption in metal PBF production by 36–49%.^37^

Finally, powder-bed AM can cause human health and toxicity concerns when operators are exposed to risks of inhaling ultrafine particles. Microplastics or metal powders that are released during production may cause adverse health effects when inhaled into human respiratory systems.^38^ Different types of polymer materials emit ultrafine particles under 100 nm, and the particle emission rate between 10^6^ and 10^12^ per minute is observed in material extrusion processes.^39^ Graff et al., Ljunggren et al., and Noskov et al. found that AM production workers are exposed to metal nanoparticles as small as 1–2 μm.^40−42^ Pre- and postprocessing steps that cannot be automatically performed within a sealed chamber are a major source of inhalation exposure.^38^

### Production Cost Disadvantages of AM

Producing the feed material needed for AM is more costly than the feedstock for certain traditional manufacturing methods, particularly in the case of metal powders.^43^ Metal powders for powder bed fusion (PBF) are about 5 to 10 times more expensive than the raw materials required in traditional manufacturing processes.^4,10^ This is caused by two factors. First, AM feedstock is smaller than that used for traditional manufacturing, which requires additional processing steps. For example, AM metal powder is produced from a feedstock of metal ingot, billet, or wire,^28^ whereas certain traditional manufacturing methods can use an ingot, billet, or wire directly as feedstock. Second, AM processes specify that the powder size should be consistent, and only 30–50% of the powder formed from atomization meets the given conditions on size and shape.^44^

A cost-premium also exists for polymer powders used for AM. While polymer powder AM feedstock is much less expensive than metal, it can be over 30 times more costly than the feedstock for traditional polymer manufacturing methods such as injection molding.^10^ This occurs because the powder size for polymer AM is much smaller and requires tighter tolerances on size and shape compared to the granulate formation for injection molding.

**Figure 1:**
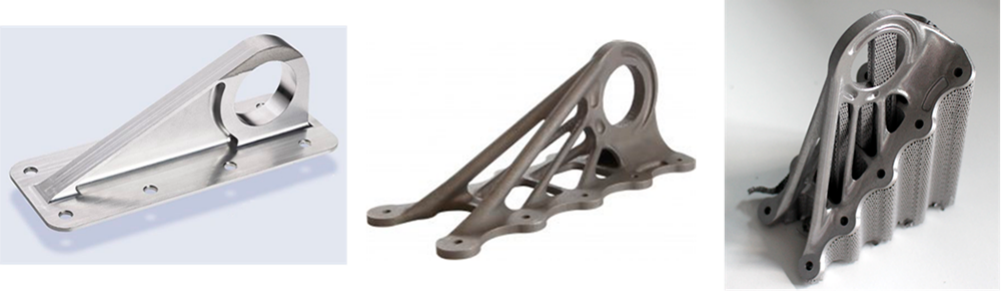
Wing bracket for Airbus A350 XWB jets: (a) original geometry for traditional manufacturing, (b) novel geometry for AM, and (c) AM part with support structures.^48^ Left images reprinted with permission from AirBus. Copyright 2017 AirBus Operations. Right image reprinted with permission from ref (48). Copyright 2022 Springer.

In addition to higher material costs, support structures that are needed to prevent distortion of an AM part before it solidifies raises production costs. For most AM processes, 3D geometries with overhangs, bridges, or holes require support structures to be built below these features simultaneously with building the part. As shown in Figure 1, these support structures increase the amount of material use, often significantly above the material in the final part. They then must be removed from the object through milling, filing, cutting, or other operations that can be time-consuming, substantially increasing production costs. In many cases, support structures typically result in wasted feedstock material as they are not reusable and have to be discarded after removal if not recyclable.^45^ In Kantareddy et al., the support structures increased production costs by 172%.^46^

Another driver of AM production costs relative to traditional manufacturing methods are postprocessing steps. The removal of support structures and the layer-by-layer build process causes AM parts to have rougher surfaces than many traditional manufactured processes.^49,50^ The poor surface finish can negatively affect mechanical properties such as fatigue and prevent a part from meeting tolerance specifications. Postprocessing steps, such as machining or polishing, are required to improve the surface finish, which further increase production costs.^45^

Finally, at low and medium production volumes (e.g., less than 100,000 units per year), the cost required for the AM machines are larger than CNC Mills, Crank Presses, and other traditional manufacturing machines.^9,10^ This is in large part because AM requires significantly more machine time than traditional manufacturing methods, and therefore, the per-unit machine cost is much higher. As seen in Atzeni et al., even when the production volume exceeds 100,000 parts per year, the amortized machine cost comprises about 59% of the unit cost for polymer AM parts.^10^ This contrasts with injection molded parts, in which machine cost comprises 2.1% of unit costs.^10^ Because of the high machine costs, the extent to which parts are packed in the machine volume is an important decision in AM and must be balanced with the risks of build failure such as postbuild part rejection, material failure, or outright build failure due to parts being packed too closely together.^51^

The highest cost machine components are those that heat and fuse the material, for example, the lasers and scanner systems.^52,53^ These components are already produced at large volumes for other industries, such as semiconductors and microprocessing,^52,54^ and so are not likely to benefit from cost reductions from economies of scale as AM production increases.

AM’s high machine costs and slow production rates call into question whether the technology can cost-effectively create local production of customized products as was previously envisioned.^53^ A recent analysis found that, while AM could theoretically be used to create distributed production, centralized manufacturing is still economically preferable because the AM machine costs are too costly and the AM process is too slow using current AM processes.^53^

## Does AM Reduce the Environmental Impacts and Costs of Mass Manufacturing?

To assess the environmental and economic impact of AM, we conducted a systematic review of comparative studies of AM and traditional manufacturing processes with respect to environmental and economic impacts. The objectives of the review were to assess literature that (1) directly compared AM and traditional manufacturing on the same part, (2) included quantitative assessment of the environmental and/or economic impacts, (3) was peer-reviewed, and (4) was published (relatively) recently so that the findings are relevant to contemporary AM capabilities. Based on these goals, the procedure for article collection and selection was first codified and then performed as follows. First, articles were collected from Scopus and Web of Science that were published since 1990 with titles or abstracts that include “additive manufacturing” or “3D printing” as well as one of the following keywords or phrases: “environmental”, “life cycle assessment”, “LCA”, “sustainability”, “sustainable”, “economic”, “cost”, or “business”. We then narrowed this set based on the criteria that the article must include a quantitative comparison of the economic costs or the environmental inventories or impacts between AM and traditional manufacturing. This eliminated papers like Conner et al., who provided a qualitative assessment of AM.^55^ From the first step of the collection procedure, we found over 2000 journal and conference papers. In the second step, this set was narrowed to 28 articles that met the criteria.

Tables 1 and 2 review comparisons of environmental impacts and production costs of AM and traditional manufacturing from the literature review. Injection molding and CNC machining are the most common manufacturing processes compared to AM in the literature because the market size of injection molding and CNC machining is larger than other manufacturing methods of plastic and metal products.^56,57^ The specific AM process chosen in each study was selected based on requirements for material, size, geometry, and functionality of printed parts and include powder-fed DED, wire-fed DED, PBF-DMLS, PBF-SLM, PBF-EBM, Binder Jetting (BJ), material extrusion, PolyJet, SLS, and vat photopolymerization. The parts studied range from small parts that are 1 in. (25.7 mm) in length to large industrial parts that are 3.2 feet (980 mm) long. The parts are produced from a variety of polymers and metals.

**Table 1:**
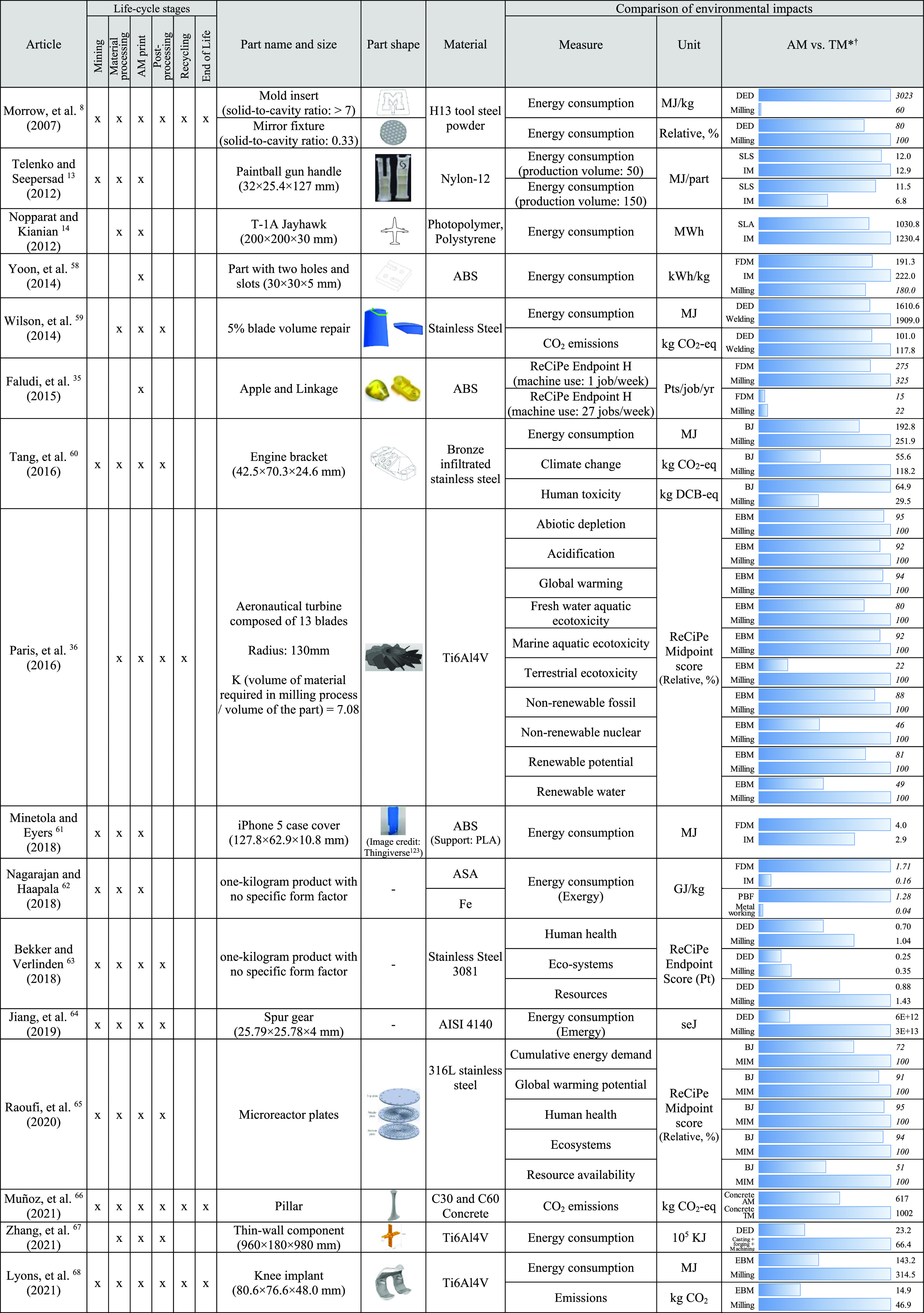
Comparison of Environmental Impacts between Additive Manufacturing and Traditional Manufacturing^a^

a Morrow et al.^8^ Figures: Reprinted with permission from Elsevier. Copyright 2007 Elsevier. Telenko and Seepersad^13^ Figure: Reprinted with permission from Emerald Publishing Limited. Copyright 2012 Emerald Publishing Limited. Wilson et al.^59^ Figure: Reprinted with permission from Elsevier. Copyright 2014 Elsevier. Faludi et al.^35^ Figure: Reprinted with permission from Emerald Publishing Limited. Copyright 2015 Emerald Publishing Limited. Tang et al.^60^ Figure: Reprinted with permission from Elsevier. Copyright 2016 Elsevier. Paris et al.^36^ Figure: Reprinted with permission from Elsevier. Copyright 2016 Elsevier. Minetola and Eyers^61^ Figure: Reprinted with permission from Thingiverse. Copyright 2012 Thingiverse. Raoufi et al.^65^ Figure: Reprinted with permission from Elsevier. Copyright 2020 Elsevier. Muñoz et al.^66^ Figure: Reprinted with permission from Springer. Copyright 2021 Springer. Zhang et al.^67^ Figure: Reprinted with permission from Elsevier. Copyright 2021 Elsevier. Lyons et al.^68^ Figure: Reprinted with permission from Springer. Copyright 2021 Springer.

* The maximum length of the bar is set to the largest value in each comparison group.

† The values in italic mean approximate values read from the graphs in the references.

**Table 2:**
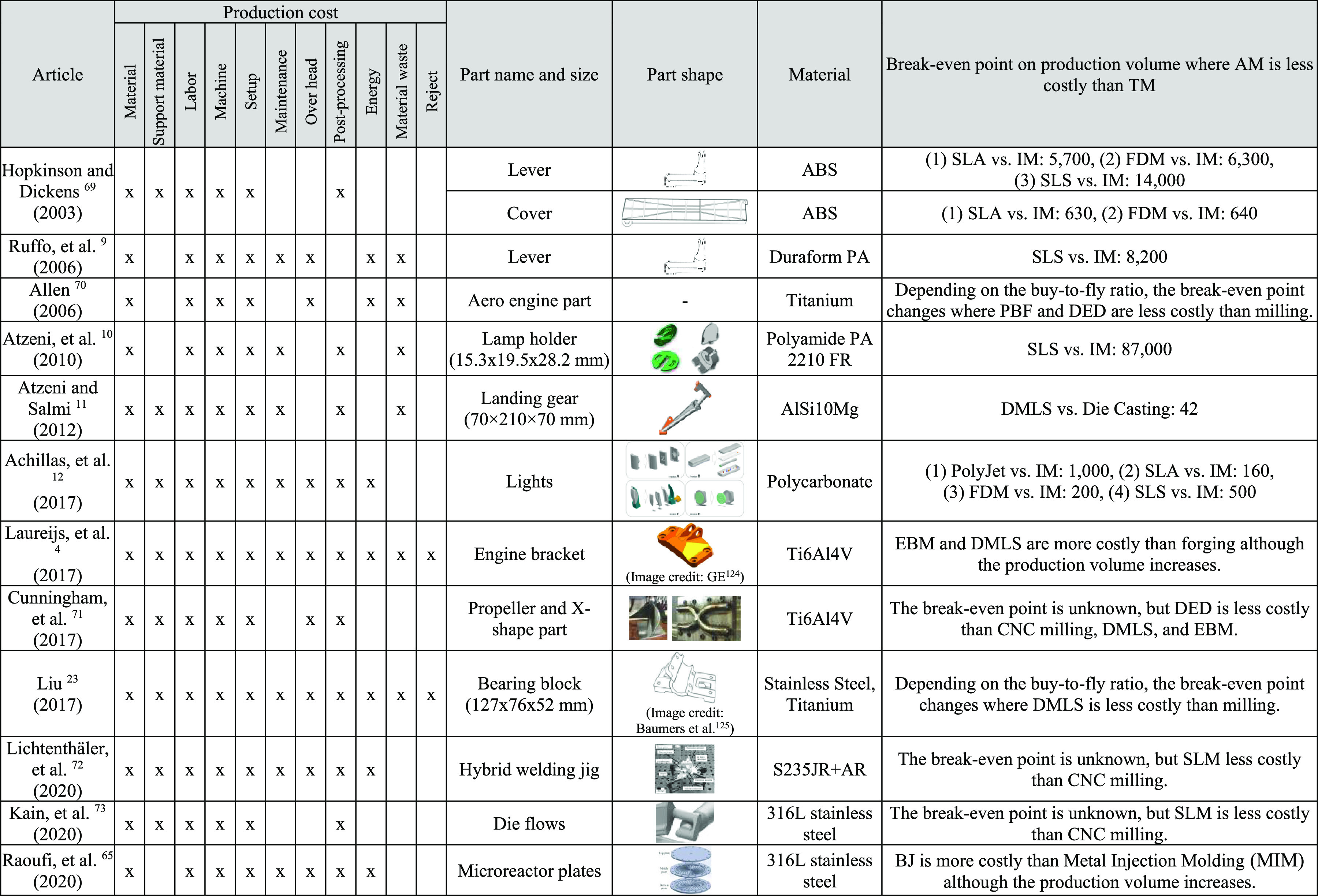
Comparison of Economic Impacts between Additive Manufacturing and Traditional Manufacturing^a^

a Atzeni et al.^10^ Figures: Reprinted with permission from Emerald Publishing Limited. Copyright 2010 Emerald Publishing Limited. Atzeni and Salmi^11^ Figure: Reprinted with permission from Springer Nature. Copyright 2012 Springer Nature. Achillas et al.^12^ Figures: Reprinted with permission from Taylor & Francis. Copyright 2017 Taylor & Francis. Laureijs et al.^4^ Figure: Reprinted with permission from GrabCAD. Copyright 2017 GrabCAD. Cunningham et al.^71^ Figures: Reprinted with permission from Elsevier. Copyright 2017 Elsevier. Liu^23^ Figure: Reprinted with permission from John Wiley and Sons. Copyright 2017 John Wiley and Sons. Lichtenthäler et al.^72^ Figure: Reprinted with permission from Springer Nature. Copyright 2020 Springer Nature. Kain et al.^73^ Figure: Reprinted with permission from Elsevier. Copyright 2020 Elsevier. Raoufi et al.^65^ Figure: Reprinted with permission from Elsevier. Copyright 2020 Elsevier.

A total of 16 studies analyzed the environmental inventories or impacts of a part when it is produced using AM in comparison to when it is produced using traditional manufacturing methods.^8,13,14,35,36,58−68^ Several of these studies (6 in total) excluded material extraction and/or end-of-life recycling or disposal in the study scope. Most of these studies compared production of the same part made with the same material for both the AM and traditional manufacturing case and accounted for differences in material scrap by using either total material used or the embodied energy and/or CO_2_ emissions in total material inputs as an indicator. As a result, we would expect the exclusion of material extraction and end-of-life to minimally affect the conclusions of these comparative studies. Ten of the studies excluded material production and/or postprocessing steps from the study scope. Unlike material extraction and end-of-life, these stages differ substantially across AM and traditional manufacturing processes. AM often requires additional postprocessing steps, such as hot isostatic pressing to relieve residual stress.^4^ In addition, the material feedstock for AM is generally more energy intensive to produce than that of traditional processes such as injection molding or milling.^7,13^ As a result, analyses that omit material production and/or postprocessing steps from the study scope are likely to underestimate the comparative lifecycle environmental impacts of AM. In addition to reviewing the findings across all studies, we investigate whether excluding the studies that omit material production or postprocessing steps affects the review but do not find that it changes the findings presented below (see the Supporting Information for further details).

The majority of the studies take a lifecycle inventory approach focusing on energy consumption and/or CO_2_-eq emissions. Bekker and Verlinden and Faludi et al. assess end-point environmental indicators, weighing across 10 impact categories following ReCiPe.^35,63^ Paris et al. and Raoufi et al. use midpoint indicators following ReCiPe.^36,65^ Of the 6 studies that assessed multiple environmental indicators, 5 found consistent results in terms of which manufacturing process had lower environmental inventories or impacts across all indicator categories. The one exception is Tang et al., which found that although BJ had lower energy consumption and CO_2_-eq emissions compared to milling, it had a higher effect on human toxicity (in terms of kg DCB-eq) because BJ uses large amounts of bronze, which has higher toxicity impacts during extraction.^60^ While other studies found that AM had lower human health or toxicity impacts compared to traditional manufacturing methods, it is important to note that they did not account for exposure of production workers to inhalation of ultrafine particles, which may significantly affect health effects, particularly when measures are not taken in the manufacturing facility to mitigate these risks.^38^ (See the Supporting Information for a more in-depth discussion.)

A total of 12 studies analyzed the production costs of a part when it is produced using AM in comparison to when it is produced using traditional manufacturing methods.^4,9−12,23,65,69−73^ All include part material, labor, setup, and machine costs in their production cost models. Laureijs et al. and Liu are the most comprehensive in terms of additionally accounting for support material, postprocessing steps, rejected parts, material waste, labor, maintenance, and overhead costs.^4,23^ Out of the 12 studies, 7 do not include maintenance or overhead costs, which may modestly underestimate the relative costs of AM compared to traditional manufacturing due to the longer machine time required for AM. Four of the studies exclude support material and/or post processing steps, which may significantly underestimate the costs of AM. In identifying the range of break-even production volumes where AM is the lower cost alternative, the upper bound was determined from a study that did not include support structures, and therefore, the upper bound should be interpreted as an estimate most appropriate for part geometries and manufacturing practices that require little to no support structures. Further details about the methods, scope, and findings of the individual studies are discussed in the Supporting Information.

## Cases Where AM Is Economically and Environmentally Preferable

### When Production Volumes Are Low

Because injection molding requires tooling that is unnecessary in AM, AM has lower environmental impacts when production volumes are very low. Telenko and Seepersad found that using SLS to produce a paintball gun handle had lower energy consumption at a volume of 50 units per year.^13^ However, when production volume is raised to 150 units per year, injection molding results in lower embodied energy of the part. This is because the SLS process consumes more energy than injection molding, and the energy intensity of the tooling is spread over a larger number of parts. The specific “break even” production volume at which injection molding will have lower lifecycle energy consumption or GHG emissions than AM depends on the part geometry and the specific AM process. For example, London et al. found that the multi jet fusion (MJF) process is less energy intensive than SLS, resulting in a break-even production volume with respect to GHG emissions of 700 for producing a LCD screen using MJF compared to injection molding.^74^

The elimination of tooling also leads to lower production costs using AM rather than injection molding when running smaller volume or customized production.^9,12,69^ As illustrated in Table 2, the break-even point is between 40 and 87,000 depending on the AM process, the part geometry and material, and the presence of support structures. For higher production volumes, AM is less cost-effective than injection molding. Hopkinson and Dicknes found that producing a cover made of ABS using stereolithography (SLA) or fused deposition modeling (FDM) was cheaper than injection molding if the production volume was lower than 630–640 units.^69^ At the other end of the scale, Atzeni et al. found that producing a lamp holder made of PA 2210 FR using SLS (with no support structures) was cheaper than injection molding up to 73,000–87,000 units.^10^ An economic comparison of PBF and CNC milling for bearing block production shows that PBF is less costly than CNC milling when the production volume is less than 5,000, but the break-even point shifts depending on part geometry.^23^

### When Traditional Parts Have High Material Waste

Tables 1 and 2 show that the geometries of parts affect the environmental impacts and production costs of AM and traditional manufacturing. In certain cases, AM can offer lower environmental impacts and lower costs at high production volumes when the part would have high material waste if traditionally manufactured. Nopparat and Kianian found that AM results in parts with lower embodied energy than injection molding if the rate of material waste in injection molding is higher than AM due to the large and complex runner system for injection molding.^14^ In their case, a scale model of a T-1A Jayhawk produced at 500,000 units per year by injection molding consumed 1,230 MWh of energy over the material processing and manufacturing lifecycle stages whereas producing the parts from SLA consumed 1,030 MWh. The lower energy consumption for AM in this case is because the part is small and so a smaller volume of support structures is required during AM, whereas many runners are required for injection molding that leads to relatively large material waste and high runner material cost.^75^

When AM is compared to manufacturing methods that produce a part from stock material, such as machining or forging, the “solid to envelope” ratio can be used to judge material waste.^76^ The solid-to-envelope ratio, also referred to as the solid-to-cavity volume, is the ratio of final part volume to the empty volume within the bounding box of a part. Thus, the ratio is an estimate of how much material is wasted in the manufacturing process. In aerospace, the inverse of the solid-to-envelope ratio is used as a common metric, called the buy-to-fly ratio (BTF).^8^ In aerospace applications, the average BTF ratio is typically lower than 1:10, meaning less than 10% of raw materials remain in the final parts.^7,8^

Paris et al. used the ratio of the volume of material required in the milling process to part volume (i.e., the inverse of the solid-to-envelope ratio) to characterize parts.^36^ As seen in Table 1, when the ratio is over 7, PBF has lower environmental impacts than CNC milling for each of the following ten impact categories: abiotic depletion, acidification, global warming, fresh water aquatic ecotoxicity, marine aquatic ecotoxicity, terrestrial ecotoxicity, nonrenewable fossil consumption, nonrenewable nuclear consumption, renewable potential, and renewable water. For example, they found that the environmental impacts of using EBM-PBF to produce aeronautical turbines are reduced by 5–51% compared to milling when the stock to part ratio is over 7. Morrow et al. find comparable results: a solid-to-cavity volume ratio of 1:7 leads to higher energy consumption using DED than CNC milling, whereas when the ratio is 1:3, DED has lower energy consumption than milling.^8^

Similar results are found with respect to the cost-effectiveness of AM. As shown in Table 2, Allen found that using current capabilities of PBF and DED had lower production costs than milling components with a solid-to-envelope ratio of about 1:12.^70^ Allen projected that this ratio could be lowered to about 1:3 for future additive manufacturing systems with improved laser power conversion efficiency, faster deposition rate (e.g., 2 kg/h), powder usage efficiency, and lower powder cost (e.g., 10–20% of current powder cost).^70^

### When AM Enables Use-Phase Performance Advantages

When AM can enable novel geometry that reduces lifecycle environmental impacts and costs through the product’s use, these may offset higher production impacts and costs. A number of applications of AM for light-weighting aircraft and automotive components have found that they reduce lifecycle environmental impacts and costs because of reduced fuel consumption during the product’s use.^4,7,77,78^ Huang et al. showed that using AM to produce a range of aircraft components across the U.S. commercial aircraft fleet could lead to a reduction of 70–173 million GJ/year of energy use by 2050, 95–98% of which is due to use-phase fuel savings by producing AM components that are between 5% and 95% lighter weight than their traditionally manufactured counterparts.^7^ Laureijs et al. estimated that producing aircraft engine brackets with EBM and DMLS as opposed to forging leads to reductions in aircraft fuel cost that outweigh the higher production costs because it enables the parts to be 80% lighter.^4^

In addition to producing lightweight parts, AM can also enable use-phase energy savings by enabling higher performance components that are used in energy generation. Recently, GE optimized the combustion system of the air-cooled H-class gas turbine by using metal AM, and the efficiency of the gas turbine increased from 63.7% to 64.0% (which is a meaningful increase for gas turbines). GE announced that the gas turbines can be used by over 70 cycle power plants and the increased efficiency of the turbine will lead to millions in fuel savings for customers globally.^79^

### When Supply Chains Can Be Shortened

AM can potentially shorten the length of supply chains by eliminating intermediate production steps.^20,53,80,81^ In cases where this supply chain simplification causes sufficient reduction of the environmental impacts and costs associated with transportation and supplier operations, it can tip the scales to make AM the environmentally and economically preferred alternative. For example, Airbus transports raw aluminum from Pittsburgh to Taiwan to produce the composite panels of the A320 aircraft. The composite panels are then transported to Toulouse, France and assembled into the final product. When AM is employed, on the other hand, the raw materials are transported directly to Toulouse to produce the final products. If the parts of the A320 are produced through AM in 2050, it is expected to reduce transport energy to 1.22 PJ/year.^20^

## Future Developments That Could Change the Equation for AM

Under the aforementioned cases, AM is currently environmentally and economically preferred to traditional manufacturing. In other cases, AM has higher environmental impacts and costs because of expensive and energy intensive material inputs, additional support structures and postprocessing steps to remove the supports, and slow production rates, particularly for high production volumes. For AM to be a sustainable alternative for mass manufacturing, technological advances are needed in computer-aided design, materials production, and use of materials in AM processes. We review recent research in these areas and identify advances that could make it the lower cost and environmental impact alternative at higher production volumes.

### Topology Optimization

Topology optimization can significantly reduce the solid-to-envelope ratios for AM parts and support structures.^82−85^ Commonly used topology optimization methods can minimize the volume of material used while maintaining stiffness and compliance constraints^86,87^ or minimizing the compliance while maintaining constraints on volume fraction.^83^ Recent advances have allowed optimization of porous infills, such as honeycomb,^88^ grid-patterns,^89^ variable-density periodic lattice,^90^ or rhombic cells^91^ to further reduce material use.

Huang et al. reviewed topology optimized AM components in aerospace and found that the AM parts reduce material use by 35–65% compared to their traditionally manufactured counterparts.^7^ This reduces material costs and has a direct benefit on machine costs as well: less material means less build time, which lowers machine costs. The energy consumption of the optimized AM part was also reduced by 59–91%. For example, the weight of an A320 nacelle hinge bracket for AM production was reduced from 918 to 326 g.^92^ These material reductions lead to reductions in embodied energy of the part as well as manufacturing time, postprocessing steps, and transportation throughout the supply chain.

**Figure 2:**
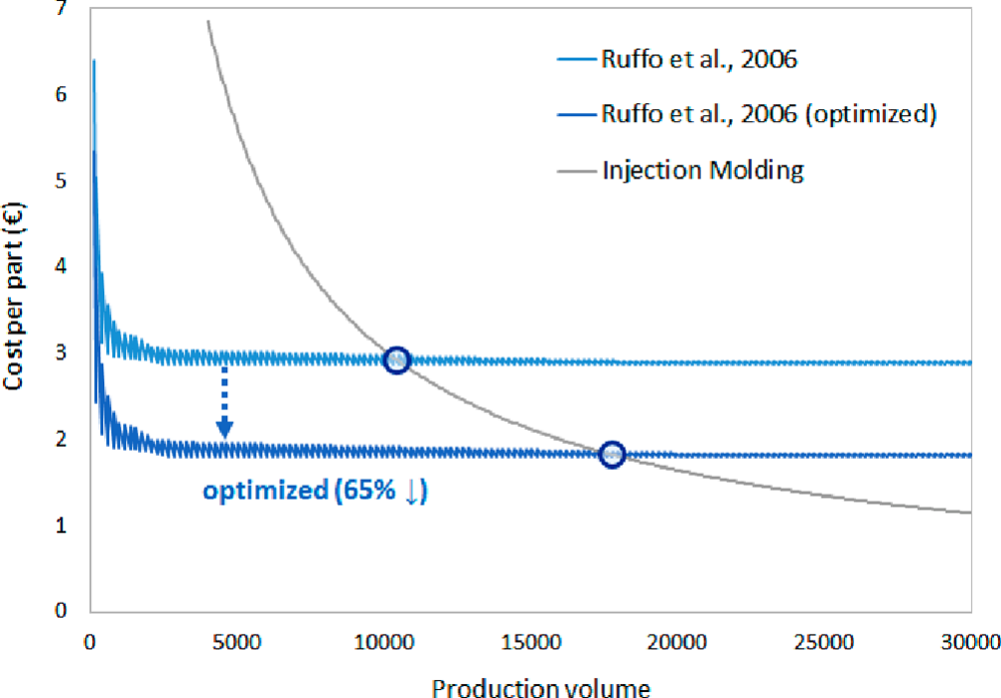
Impact of topology optimization on production costs.

As shown in Figure 2, if we assume that the weight is reduced by up to 65% through topology optimization of the product,^7^ it increases the break-even production volume between AM and traditionally manufacturing methods. For example, for the cost model defined by Ruffo et al., the break-even point shifts from 10,500 to 18,000 parts.^9^

**Figure 3:**
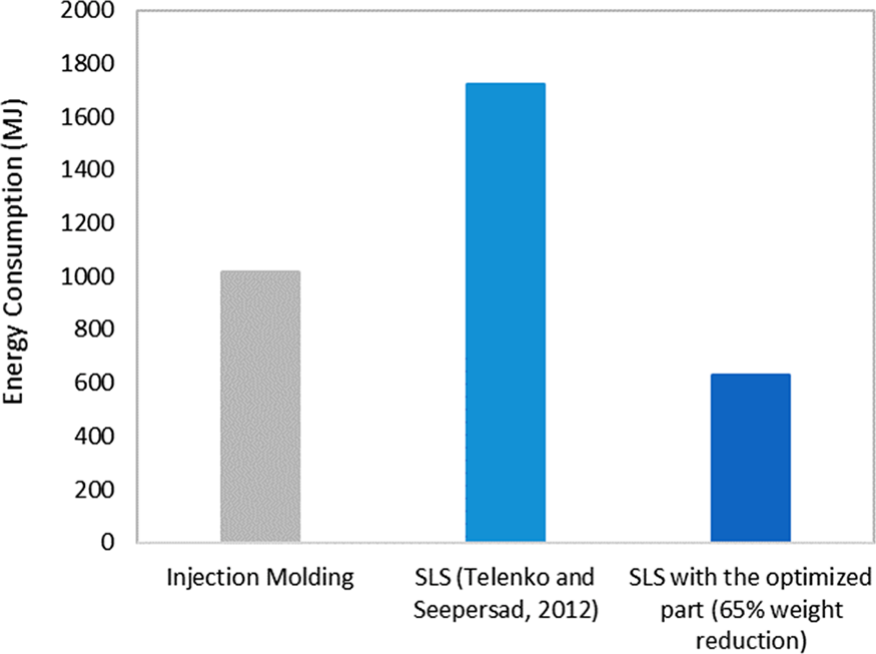
Impact of topology optimization on lifecycle energy use.

Similarly, topology optimization of products for AM would shift the break-even production volume for which AM has lower environmental impacts than traditional manufacturing processes. Assuming the same 65% weight savings from topology optimization, we calculate that the lifecycle energy consumption of a nylon paintball gun handle from Telenko and Seepersad’s LCI study reduces such that SLS has lower lifecycle energy consumption than injection molding at a production volume of 150 per year (Figure 3).^13^ In contrast, the break-even point using the original part weight was only approximately 50 per year.

### Dissolvable Support Structures

Further development and adoption of multimaterial AM has the potential to significantly reduce the environmental impacts and production costs of AM.^45,49^ Hopkins et al., Ni et al., Hildreth et al., and Lefky et al. showed that the production costs in AM can be reduced by selecting support materials that are dissolvable or easily removed.^49,93−95^ For polymer AM, the dissolvable support structures such as PVA (poly(vinyl alcohol)) and HIPS (high-impact polystyrene) can be utilized; for instance, PVA is quickly dissolved in water, so using water-soluble materials can contribute to significantly reducing postprocessing costs to remove support structures.^45,94^ For metal AM, Hildreth et al. suggested using dissolvable carbon steel supports for the powder-fed DED process, which would reduce the use of the more energy-intensive powder feedstock.^95^ Lefky et al. showed that the use of dissoluble support structures could also save labor and machining costs required to remove support structures (e.g., $900-$4,000 for interlocking stainless steel rings, depending on part complexity).^49^

### Alternatives for Support Structures

AM processes that allow for reusable alternatives for support structures can cut down both the environmental impacts and costs of these materials. For example, Xu et al. developed a programmable build platform with dynamically controlled metal pins that can support plastic parts printed using material extrusion.^96^ They found that the reusable pins reduce supporting materials by 64.7% on average (ranging from 22.6% to 100%) and reduce production time by 63% on average compared to conventional material extrusion processes.^96^

Recent research using support baths for polymer or metal AM has been conducted by Hinton et al. and Yu et al.^97,98^ Support baths are reusable and can act as support structures by stabilizing printing parts. Hinton et al. utilized a hydrophilic Carbopol support bath to print polydimethylsiloxane (PDMS) polymers, and Yu et al. suggested a supporting method using the self-healing hydrogel to print liquid metals into macroscopic 3D structures.^97,98^

### AM Processes That Do Not Require Support Structures

Some AM processes such as BJ do not require support structures, but BJ needs “setters” that are used to prevent the deformation under the part’s weight during sintering.^99^ The setters should be carefully designed to be compatible with part geometry because of part shrinkage issues during high-temperature sintering processes, but the setters can be made of materials such as ceramics that are less energy intensive and costly than metal AM feedstock, thus giving BJ an environmental and economic advantage compared to metal AM processes that require significant support structures.^99^

### Self-supporting Structures

**Figure 4:**
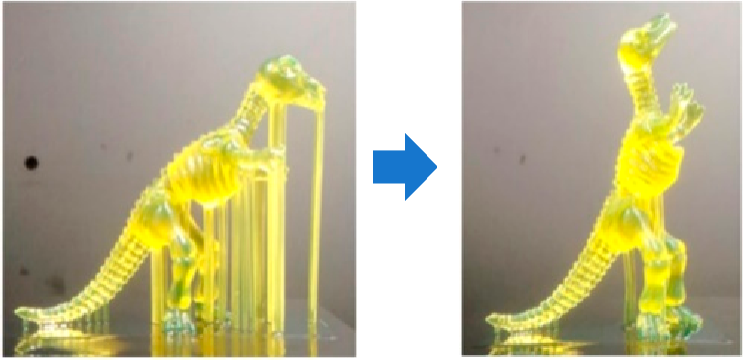
Example of self-supporting structures from Hu et al.^101^ Reprinted with permission from ref (101). Copyright 2015 Elsevier.

Methods have been developed to produce part geometries that are self-supporting, thus eliminating the environmental impacts and costs associated with support structures. Leary et al. suggested an automated method to modify topologically optimal geometries for enabling support-free additive manufacturing in the case of FDM, and they showed that the total material consumption and build time of self-supporting structures are less than those of topologically optimal geometries without self-supporting in cantilever beam examples.^100^ The build time and material consumption were reduced by 54.4% (from 5.7 to 2.6 h) and 38.8% (from 89.7 to 54.9 cm^3^), respectively. Hu et al. developed a method of modifying a given part topology by adjusting the angles and shapes of features to meet minimum overhang constraints to be self-supporting.^101^Figure 4 shows the optimized geometry and build orientation of the printed part.

Recent studies have extended self-supporting algorithms to incorporate overhang constraints directly into topology optimization.^102−104^ These methods create density filters that enforce the overhang constraint at every iteration of the topology optimization. They accomplish this by defining the density of any element as a function of the densities of other elements that can support it based on finite element meshes,^102^ spatial density gradients,^104^ or searching for elements in a defined support region.^103^ An advantage of these methods is they find the self-supporting geometry that minimizes production cost, time, and/or structural compliance or maximize eigenfrequency, rather than relying on posthoc modifications to the geometry.^102−105^

### Advances in Material Production

Reducing the energy consumption and costs to produce AM materials would significantly improve AM’s environmental and economic competitiveness with traditional manufacturing processes. This is particularly an issue with powder feedstock from high-cost metals such as titanium, where materials can account for 33–58% of lifecycle energy consumption and 20–77% of costs.^7,23,68,72,106^ For polymer AM, although materials make up a smaller percentage of lifecycle energy consumption and production costs, AM feedstock can still be 1.4–2.1 times the energy intensity and 3.6–15.4 times the cost of injection molding feedstock.^10,13,61,107^

The minerals from which titanium is processed contain large amounts of oxygen, which is reduced for smelting before processing the titanium as a powder or wire stock for AM. Excess oxygen retained in the titanium feedstock compromises ductility and fracture toughness and is more common in powder than the larger stock required for machining, casting, or forging.^108,109^ This contributes to the high cost of AM feed stock as the yield for titanium powder is low. Potential developments of smelting processes may lower these costs by reducing the oxygen content in titanium powder and producing highly spherical powders. The Armstrong process allows titanium tetrachloride to be reduced to titanium at low temperatures and results in relatively low amounts of oxygen in powders.^110,111^ Because of the lower temperatures and higher powder yield, the process reduces the energy intensity and cost of titanium feed stock for AM. Peter et al. found that energy consumption was cut by 53.4% from 355 to 165 MBtu/ton.^110^

**Figure 5:**
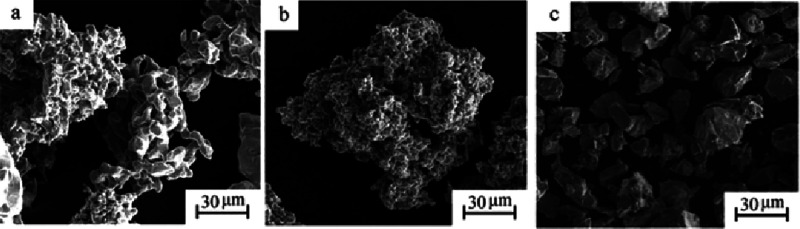
Micrographs of Ti-6Al-4 V powders using the (a) Kroll process, (b) Armstrong process, and (c) HDH process.^114^ Reprinted with permission from ref (114). Copyright 2018 Maney Publishing.

The Hydride-Dehydride (HDH) process utilizes the chemically reversible reaction between titanium and hydrogen.^112,113^ This process hydrogenates titanium feedstock in atmospheric hydrogen pressure, and the titanium hydride is milled into powder. As shown in Figure 5, compared to other methods using gas or plasma atomization, the HDH process can produce low-cost titanium powders even though the process still has some issues with irregular particle shapes.^112^

Fueling gas and plasma atomization processes with less carbon intensive sources of energy would reduce the lifecycle CO_2_ emissions of AM relative to traditional manufacturing methods such as machining. As decarbonization of industrial processes evolves, switching to alternative sources of energy, such as hydrogen, biomass, or fossil fuels with carbon capture and sequestration may be possible.

Advances in polymer AM feedstock would also help to improve the competitiveness of AM compared to traditional manufacturing methods such as injection molding. Common feedstock for polymer AM includes liquid-type materials (e.g., for vat photopolymerization), powder types (e.g., for SLS), and filament types for material extrusion. In the case of SLS powder feedstock, the cost is over 15 times that of injection molding feedstock.^10^ Current requirements for SLS polymer powders are that they are spherical and have distributed particle size, flowability, infrared absorption, low zero viscosity, and low surface tension, which increase the cost of powder production.^115,116^ Due to the plastic or viscoelastic material behavior of polymers, grinding to produce polymer powders is costly and energy intensive.^115^ Developing advanced powder production methods such as wet grinding, rounding, and dry coating to improve powder flowability will lead to cost reduction and energy savings of SLS feedstock.^115^

Increasing the recycled material in AM feedstock could lower the production costs and environmental impacts of AM if future advances are made to limit the effects of recycled content on material properties. Whether the quality and flow characteristics of AM feedstock and the resulting mechanical properties of the AM part are degraded depends on many factors, including the specific material, the AM modality, and processing parameters. In certain contexts, the tensile strength and ultimate strength of AM parts have been found to degrade more quickly with the number of recycling cycles faster than for injection molded parts.^117^ Recent research has investigated how altering the blend of materials and additives can mitigate this degradation of mechanical properties.^118^ Another potentially promising route is to include recycled content in the gas atomization process during powder production, which in the case of AISI 316L powder was found to slightly improve tensile strength and maintain the same processability as compared with primary powder.^119^

### Reducing AM Machine Build Time and Energy Use

Because of their slower build times, AM machines tend to result in higher energy consumption and CO_2_ emissions than conventional manufacturing machines to produce their parts, and more machines are needed to produce parts at the same rate, increasing overall machine costs. Laureijs et al. showed that the metal AM machine cost accounts for 20–44% of amortized production costs for DMLS and EBM parts.^4^

Powering AM facilities with lower-carbon sources of electricity would help to reduce the CO_2_ emissions of AM. If AM facilities are sited in locations where the marginal CO_2_ emissions of electricity are lower than facilities producing tooling for traditional manufacturing methods such as casting and injection molding, this could increase the production volume for which AM has lower lifecycle CO_2_ emissions relative to the traditional manufacturing method.

Increasing build speed would serve to reduce CO_2_ emissions, energy use, and machine costs associated with AM. One potential route to reducing AM build time is to use multilaser AM machines, which can build multiple parts at once and thus reduce total build times and energy use of producing the parts. Recently, SLM solution launched a 12-laser metal PBF machine, which is around 20 times faster than a single-laser machine and results in significantly less energy consumption and capital costs than 12 single-laser machines.^120^ Whether multilaser machines reduce overall energy consumption and costs depends on the number of lasers, laser power, part size, and process parameters.^121^ Additionally, using multiple moving lasers during build creates unknown effects on microstructural properties and defect rates that could undermine the advantages.^122^ Further development is needed to address these effects and demonstrate that total production time and energy use accounting for rejected parts is improved in practice.

## Conclusions

While AM parts tend to have lower weight and material use in their final form, they have higher environmental impacts and costs associated with more hidden aspects of production that are not apparent in the final part, such as support structures, postprocessing, and expensive and energy-intensive feedstocks. These impacts are not caused by economies of scale and therefore are not likely to be addressed by increasing use of AM. We review comparative studies of the environmental impacts and production costs of AM relative to traditional manufacturing processes across a wide variety of product applications, materials, and part geometries. We find that AM has lower environmental impacts when production volumes are very low (approximately 1,000 parts per year or less) unless the part geometry has a solid-to-envelope ratio of 1:7 or above. The higher relative environmental impacts and production costs of AM at large production volumes can be offset by one or more of the following factors: (1) the parts are small and have geometries with high material waste in traditional manufacturing; (2) AM offers performance advantages that reduce lifecycle environmental impacts and costs; (3) portions of the supply chain are able to be eliminated, reducing the environmental impacts and costs associated with transportation and facility operations. Future advances in material production, topology optimization, reusable support structures, self-supporting structures, and AM process improvements that speed up printing time while mitigating defects could potentially tip the scales so that AM has lower environmental and economic impacts than traditional manufacturing in a wider set of contexts. Concentrating AM research and development on these goals would serve to improve both the economic and environmental advantages of AM in a wider set of contexts.

The Critical Review also highlights three important considerations for studies comparing the environmental or economic impacts of AM in comparison to traditional manufacturing methods. First, when assessing whether a part should be produced using AM, the study should use the redesigned part with the geometry optimized for AM as the comparison part. Second, material production, support structures, and postprocessing steps such as removal of support structures, heat treatment, and any necessary machining or surface treatment steps should be included in the analysis to avoid significantly underestimating the environmental impacts and/or production costs of AM. Third, when the application involves significant energy use during the use phase that could be affected by the part geometry or manufacturing process, LCA and lifecycle costing approaches should be used that include the use phase as well as upstream lifecycle stages. Finally, the review shows that the type of manufacturing process that has lower lifecycle energy consumption and CO_2_ emissions tends to also have lower impacts with respect to other ecosystem and resource availability indicators, such as water use, acidification, and aquatic and terrestrial ecotoxicity. However, AM may have higher human toxicity impacts even when it has lower impacts across all other categories. Based on the review, we recommend that comparative studies of AM and traditional manufacturing methods include at least lifecycle energy consumption and/or CO_2_ emissions, human toxicity, and lifecycle cost as comparison metrics.

## Supporting Information Available

Additional details about the methods, scope, and data sources for the studies reviewed (PDF)
