# Supplementary material for: Is Additive Manufacturing an Environmentally and Economically Preferred Alternative for Mass Production?
Source: Environ Sci Technol. 2023 Apr 17;57(16):6373–86. doi: 10.1021/acs.est.2c04927 (PMC10134501; doi:10.1021/acs.est.2c04927)
Supplement: Supplementary file 1 — es2c04927_si_001.pdf [file es2c04927_si_001.pdf]

Supporting Information for

Is additive manufacturing an environmentally and economically preferred alternative for mass production?

Sangjin Jung, Levent Burak Kara, Zhenguo Nie, Timothy W. Simpson, and Kate S. Whitefoot

Number of Pages: 7

Number of Figures: 0

Number of Tables: 2

Review of Comparative Environmental LCA Study Methods and Scope

A total of 16 studies analyzed the environmental inventories or impacts of a part when it is produced using AM in comparison to when it is produced using traditional manufacturing methods. All of these studies used a comparative life cycle analysis (LCA) approach. Out of the 16 studies, 7 explicitly report following the ISO 14040 standards for LCA. Table S1 provides details regarding the functional unit, data sources, and methods used for each study.

Several of the studies (6 in total) excluded material extraction and/or end-of-life recycling or disposal in scope. Most of these studies compared identical parts made from identical material that would share the same mining processes and end-of-life. Further, most of them accounted for differences in material scrap across AM and traditional manufacturing processes by using either total material used or the embodied energy and/or CO_2 emissions in total material inputs as an indicator. As a result, we would expect the exclusion of material extraction and end-of-life to minimally affect the conclusions of these comparative studies. 10 of the studies excluded materials production and/or post-processing steps from the scope of the analysis. Unlike material extraction and end-of-life, these stages differ across AM and traditional manufacturing processes. AM often requires additional post-processing steps such as hot isostatic pressing to relieve residual stress. In addition, the material feedstock for AM is generally more energy intensive to produce than that of injection molding or milling. As a result, analyses that omit materials production and/or post-processing steps from the study scope are likely to underestimate the lifecycle environmental impacts of AM in comparison to injection molding or milling.

The studies differ in terms of their goals and the environmental indicators used. Bekker and Verlinden (2018) and Faludi et al. (2015) assess end-point environmental indicators following ReCiPe. Paris et al. (2016) and Raoufi et al. (2020) use mid-point indicators following ReCiPe. The remainder of the studies use life cycle inventories as indicators, primarily assessing energy consumption and/or CO_2-eq. emissions. Of the 6 studies that assessed multiple environmental indicators, 5 found consistent results in terms of which manufacturing process had lower environmental inventories or impacts across all indicator categories. The one exception is Tang et al., which found that although BJ had lower energy consumption and CO_2-eq. emissions compared to milling, it had a higher effect on human toxicity (in terms of kg DCB-eq.) because BJ uses large amounts of bronze, which has higher toxicity impacts during extraction (Tang et al. 2016). While other studies found that AM had lower human health or toxicity impacts compared to traditional manufacturing methods, it is important to note that they did not account for exposure of production workers to inhalation of ultrafine particles, which may significantly affect health effects, particularly when measures are not taken in the manufacturing facility to mitigate these risks (Kolb et al., 2017).

A consistent finding across the studies is that when production volumes are small (approximately 1,000 parts per year or less) and the part has a geometry with a relatively low solid-to-envelope ratio (<1:7), AM has lower energy consumption, lower greenhouse gas emissions–and for the 4 studies that investigated mid-point or end-point environmental

impact indicators–lower impacts on ecosystem and human health indicators. This finding remains even when we exclude studies that omit material production or post-processing steps from the scope of the study, which in our assessment are likely to underestimate the lifecycle environmental impacts of AM. While lifecycle environmental impacts are lower at small production volumes, it is possible that AM processes may result in higher human toxicity, especially for Binder Jetting with bronze infiltration or for other AM processes when mitigation measures are not taken to limit operator exposure to ultra-fine particles during AM production (Katz et al., 2020; Kolb et al., 2017; Tang et al. 2016).

Few of the studies addressed uncertainties in impact categories that may affect the comparison of AM with traditional manufacturing methods. It is important to note that some life cycle impact categories can have large sources of uncertainty and may have differentially larger uncertainty compared to others. This may affect the comparison of the lifecycle environmental impacts of AM compared to traditional manufacturing. For example, uncertainties associated with life cycle human toxicity impacts are high, with large sources of variation in many factors, including intake and the likelihood of developing an adverse health effect. Thus, caution should be taken when comparing the point values of human toxicity impacts across AM and traditional manufacturing methods with point values of other impact categories (e.g., energy use).

Review of Comparative Production Cost Study Methods and Scope

A total of 12 studies analyzed the production costs of a part when it is produced using AM in comparison to when it is produced using traditional manufacturing methods. 11 of these studies used a process-based cost approach that identifies the required inputs (e.g., materials, machine time, labor) for each of the production process steps and determines costs of these inputs as well as indirect costs (e.g., maintenance and overhead) that are needed to run the facility. One of the studies used an activity-based costing approach where both direct and indirect costs of production steps are determined based on the time that it takes to complete the step. Out of the 12 studies, 5 validated their models by comparing the model estimates of predicted inputs or production costs with manufacturing companies and/or an AM fabrication testbed. Table S2 provides details regarding the functional unit, data sources, and methods used for each study.

The studies differ in terms of their goals and scope. All studies include part material, machine, and setup costs in scope. Laureijs et al. (2017) and Liu (2017) are the most comprehensive in terms of additionally accounting for support material, post-processing steps, rejected parts, material waste (scrap), labor, maintenance, and overhead costs. 5 out of the 12 studies exclude energy costs from the analysis, which is a relatively small portion of total production costs. 7 of the 12 studies do not include maintenance or overhead costs, which may underestimate the relative costs of AM compared to traditional manufacturing due to the longer machine time required for AM. 4 of the studies exclude support material and/or post processing steps, which could significantly underestimate the costs of AM

because AM often requires significant additional material use for support structures and post-processing steps such as hot isostatic pressing to relieve residual stress.

A consistent finding across the studies is that when production volumes are small (between 42-87,000 per year depending on the AM process and part geometry), AM has lower production costs than traditional manufacturing processes such as casting or injection molding. The upper bound was found from a study that did not include support structures, and therefore the upper bound should be interpreted as an estimate most appropriate for part geometries and manufacturing practices that require little to no support structures. Excluding all studies that omit support structures and/or post-processing steps reduces the range of break-even production volumes between AM and traditional manufacturing methods to 42-14,000 per year. Using AM methods that have faster production speeds and parts with geometries that have a higher buy-to-fly ratio moves the break-even production volume toward the upper end of this range.

Table S1. Method characterization of articles comparing environmental impacts of AM with traditional manufacturing

Article	Method		Functional Unit	Data Used in. Analysis		
	Comparative LCI or LCA	ISO Standard Followed		Primary	Secondary	Data Sources
Morrow et al. (2007)	X		1 Mold insert and mirror fixture	X	X	Own data, U.S. Department of Energy, DTE Energy, Yule and Dunkley (1994)
Telenko and Seepersad (2012)	X		1 paintball gun handle	X	X	Own data, GaBi (PlasticEurope database), Thiriez and Gutowski (2006), Dotchev and Yussof (2009)
Nopparat and Kianian (2012)	X	14040	1 scale model kit of T-1A Jayhawk Air Force Trainer in 1:72 scales	X	X	3Delivered, Inc., Click2detail, Inventory of Carbon and Energy v1.6a, Luo et al. (1999), Dahmus and Gutowski (2004)
Yoon et al. (2014)	X		1 part with two holes and slots	X		Own data
Wilson et al. (2014)	X		1 operational turbine blade made of a nickel alloy	X	X	Own data, Ecoinvent 2, Morrow et al. (2007), Margolis et al. (1999)
Faludi et al. (2015)	X		1 “job” comprising the manufacturing of two different parts in plastic	X	X	Own data, MSDS, Diaz et al. (2011)
Tang et al. (2016)	X		1 engine bracket	X	X	Own data, Ecoinvent 3
Paris et al. (2016)	X		1 aeronautical turbine composed of 13 blades	X	X	Own data, Ecoinvent
Minetola and Eyers (2018)	X		1 Apple iPhone 5 cover	X	X	Own data, CES Edupack 2016
Nagarajan and Haapala (2018)	X	14040<br>14042	1 kg of Fe (or ASA) with no specific form factor	X	X	Own data, Ecoinvent 3
Bekker and Verlinden (2018)	X	14040	1 kg of manufactured Stainless Steel 308L in a one-off (batch size of one) production	X	X	Own data, Ecoinvent 3, CES, Idemat 2014
Jiang et al. (2019)	X		2 gears manufactured by LENS and CNC machining	X	X	Own data , GaBi, Liu et al. (2017)
Raoufi et al. (2020)	X	14040	1 full microreactor plate (one middle and one bottom plate)	X	X	Own data, Ecoinvent 3
Muñoz et al. (2021)	X	14040<br>14044	1 pillar	X	X	Own data, EXIOBASE
Zhang et al. (2021)	X		1 component	X		Own data
Lyons et al. (2021)	X	14040<br>14041	1 knee implant made from Ti-6Al-4V	X	X	Own data, CES, Le and Paris (2018)

Table S2. Method characterization of articles comparing production costs of AM with traditional manufacturing

Article	Method			Functional Unit	Data Used in Analysis			Model and/or cost estimates validated with manufacturing companies or testbeds?
	Process-based cost model	Activity-based costing	ISO 14040		Primary Data	Secondary Data	Data Collection	
Hopkinson and Dickens (2003)	X			(1) Lever (2) Cover	X		Data collected by authors	
Ruffo et al. (2006)	X			Lever		X	Hopkinson and Dickens (2003), ManRM, and Guide to Duraform	X
Allen (2006)	X			Aero engine part	X		Data collected by authors	
Atzeni et al. (2010)	X			Lamp holder	X		Data collected by authors	
Atzeni et al. (2012)	X			Landing gear	X		Data collected by authors	
Achillas et al. (2017)	X			Lights	X		Data collected by authors	
Laureijs et al. (2017)	X			Engine bracket	X		14 companies, including metal additive manufacturing product producers, material, and equipment suppliers, and university laboratories	X
Cunningham et al. (2017)		X		Propeller and X-shape part	X		Data collected by authors	X
Liu (2017)	X			Bearing block		X	Baumers et al. 2012, supplemented with data from companies	X
Lichtenthäler et al. (2020)	X			Hybrid welding jig	X		Data collected by authors, supplemented by data from Schmidt (2015), Kühn et al. (2018)	
Kain et al. (2020)	X			Die flows	X		Data collected by authors, supplemented by data from Hällgren et. al. (2016)	
Raoufi et al. (2020)	X		X	Microreactor plate (one middle and one bottom plate)	X		Data collected by authors	X

References

Achillas, C.; Tzetzis, D.; Raimondo, M. O. Alternative production strategies based on the comparison of additive and traditional manufacturing technologies. *Int J Prod Res* **2017**, *55* (12), 3497-3509.

Allen, J. An investigation into the comparative costs of additive manufacture vs. machine from solid for aero engine parts. In Cost Effective Manufacture via Net-Shape Processing, Neuilly-sur-Seine, France; 2006.

Atzeni, E.; Iuliano, L.; Minetola, P.; Salmi, A. Redesign and cost estimation of rapid manufactured plastic parts. *Rapid Prototyping Journal* **2010**, *16* (5), 308-317.

Atzeni, E.; Salmi, A. Economics of additive manufacturing for end-usable metal parts. *The International Journal of Advanced Manufacturing Technology* **2012**, *62*, 1147-1155.

Bekker, A. C. M.; Verlinden, J. C.. "Life cycle assessment of wire+ arc additive manufacturing compared to green sand casting and CNC milling in stainless steel." *Journal of Cleaner Production* **2018**, *177*, 438-447.

Dahmus, J. B. and Gutowski. T. G. (2004), “An Environmental Analysis of Machining,” *Proceedings of the 2004 ASME International Mechanical Engineering Congress and RD&D Exposition*. November 13- 19. Anaheim. California.

Diaz, N., Redelsheimer, E. and Dornfeld, D. (2011), *Energy Consumption Characterization and Reduction Strategies for Milling Machine Tool Use*, Laboratory for Manufacturing and Sustainability, University of California, Berkeley.

Dotchev, K. and Yussof, W. (2009), "Recycling of polyamide 12 based powders in the laser sintering process", *Rapid Prototyping Journal*, 15(3), 192-203.

Faludi, J.; Baumers, M.; Maskery, I.; Hague, R. Environmental impacts of selective laser melting: do printer, powder, or power dominate? *Journal of Industrial Ecology* **2017**, *21* (S1), S144-S156.

Faludi, J.; Bayley, C.; Bhogal, S.; Iribarne, M. Comparing environmental impacts of additive manufacturing vs traditional machining via life-cycle assessment. *Rapid Prototyping Journal* **2015**, *21* (1), 14-33.

Guide to Duraform Materials, 3D Systems, May 2002, DCM 8002–10004–001.

Hällgren S, Pejryd L, Ekengren J. (2016) "Additive Manufacturing and High Speed Machining - Cost Comparison of short Lead Time Manufacturing Methods*". J Procedia CIRP*, 50:384–9

Hopkinson, N.; Dickens, P. Analysis of rapid manufacturing—using layer manufacturing processes for production. *Proceedings of the Institution of Mechanical Engineers, Part C: Journal of Mechanical Engineering Science* **2003**, *217* (1), 31-39.

Kolb, T.; Schmidt, P.; Beisser, R.; Tremel, J.; Schmidt, M. Safety in additive manufacturing: Fine dust measurements for a process chain in Laser beam melting of metals. *RTeJournal-Fachforum für Rapid Technologie* **2017**, *2017* (1).

Kühn, K.D., Fritz A.H., Förster, R., and Hoffmeister, H.W. (2018) Trennen. In: FritzA(ed) Fertigungstechnik. Springer Vieweg, Berlin

Laureijs, R. E.; Roca, J. B.; Narra, S. P.; Montgomery, C.; Beuth, J. L.; Fuchs, E. R. H. Metal additive manufacturing: Cost competitive beyond low volumes. *Journal of Manufacturing Science and Engineering* **2017**, *139* (8), 081010.

Le, V. T. and Paris, H. (2018). "A life cycle assessment-based approach for evaluating the influence of total build height and batch size on the environmental performance of electron beam melting," *The International Journal of Advanced Manufacturing Technology*, 98(1), 275-288.

Liu, Z. Economic comparison of selective laser melting and conventional subtractive manufacturing processes. Northeastern University, Boston, MA, 2017.

Liu, Z., Jiang, Q., Cong, W., Li, T., and Zhang, H. C. (2018). "Comparative study for environmental performances of traditional manufacturing and directed energy deposition processes," International Journal of Environmental Science and Technology, 15(11), 2273-2282.

Luo, Y., Ji, Z., Ming, L., and Caudill, R. (1999), "Environmental Performance Analysis of Solid Freeform Fabrication Processes," *Proceedings of the 1999 IEEE International Symposium on Electronics and the Environment*, May 11-13.

Lyons, R.; Newell, A.; Ghadimi, P.; Papakostas, N. Environmental impacts of conventional and additive manufacturing for the production of Ti-6Al-4V knee implant: a life cycle approach. *The International Journal of Advanced Manufacturing Technology* **2021**, *112* (3), 787-801.

ManRM: Management, Organisation and Implementation of Rapid Manufacturing. Foresight vehicle research project developed at the Loughborough University and funded by the DTI.

Margolis, N., Jamison, K., and Dove, L. (1999), *Energy and Environmental Profile of the U.S. Metal Casting Industry*, U.S. Department of Energy, Office of Industrial Technologies, Washington, D.C.

Morrow,W.R., Qi, H., Kim, I., Mazumder, J. and Skerlos, S.J. (2007), "Environmental aspects of laser-based and conventional tool and die manufacturing," *Journal of Cleaner Production,* 15(10), 932-943.

Muñoz, I.; Alonso-Madrid, J.; Menéndez-Muñiz, M.; Uhart, M.; Canou, J.; Martin, C.; Fabritius, M.; Calvo, L.; Poudelet, L.; Cardona, R. Life cycle assessment of integrated additive–subtractive concrete 3D printing. *The International Journal of Advanced Manufacturing Technology* **2021**, *112* (7), 2149-2159.

Nagarajan, H. P. N.; Haapala, K. R. Characterizing the influence of resource-energy-exergy factors on the environmental performance of additive manufacturing systems. *Journal of Manufacturing Systems* **2018**, *48*, 87-96.

Nopparat, N.; Kianian, B. Resource Consumption of Additive Manufacturing Technology. Blekinge Institute of Technology, Karlskrona, Sweden, 2012.

Paris, H.; Mokhtarian, H.; Coatanéa, E.; Museau, M.; Ituarte, I. F. Comparative environmental impacts of additive and subtractive manufacturing technologies. *CIRP Annals* **2016**, *65* (1), 29-32.

Rännar, L. E., Glad, A., and Gustafson, C. G. (2007). "Efficient cooling with tool inserts manufactured by electron beam melting," Rapid Prototyping Journal.

Raoufi, K.; Manoharan, S.; Etheridge, T.; Paul, B. K.; Haapala, K. R. Cost and environmental impact assessment of stainless steel microreactor plates using binder jetting and metal injection molding processes. *Procedia Manufacturing* **2020**, *48*, 311-319.

Ruffo, M.; Tuck, C.; Hague, R. Cost estimation for rapid manufacturing-laser sintering production for low to medium volumes. *Proceedings of the Institution of Mechanical Engineers, Part B: Journal of Engineering Manufacture* **2006**, *220* (9), 1417-1427.

Schmidt, T. (2015) Potentialbewertung generativer Fertigungsverfahren für Leichtbauteile. Thesis (Ph.D.), Technischen Universität Hamburg-Harburg

Tang, Y.; Mak, K.; Zhao, Y. F. A framework to reduce product environmental impact through design optimization for additive manufacturing. *Journal of Cleaner Production* **2016**, *137*, 1560-1572.

Telenko, C.; Seepersad, C. C. A comparison of the energy efficiency of selective laser sintering and injection molding of nylon parts. *Rapid Prototyping Journal* **2012**, *18* (6), 472-481.

Thiriez, A. and Gutowski, T. (2006), "An environmental analysis of injection molding", *IEEE International Symposium on Electronics and the Environment*, San Francisco, CA, May 8-11.

Yule, A.J. and Dunkley, J. J. (1994), *Atomization of melts for powder production and spray deposition*, New York, NY: Oxford University Press.
